# Disaster adaptation wisdom and resilience mechanisms in the traditional settlement landscape of Mentawir Village, capital city of Nusantara, East Kalimantan

**DOI:** 10.4102/jamba.v18i1.2006

**Published:** 2026-10-05

**Authors:** I Made Geria, Retno Handini, Titi Surti Nastiti, Ni Putu Eka Juliawati, Mohammad Ruly Fauzi, Unggul Prasetyo Wibowo, Emi Purwanti, Yunianto Setiawan, I Nyoman Indra Kumara

**Affiliations:** 1 Research Center for Environmental Archaeology, Maritime Archaeology, and Cultural Sustainability, National Research and Innovation Agency, Jakarta, Indonesia; 2 Research Center for Prehistoric and History Archaeology, National Research and Innovation Agency, Jakarta, Indonesia; 3 Research Center for Archaeometry, National Research and Innovation Agency, Jakarta, Indonesia; 4 Bandung Museum of Geology, Ministry of Energy and Mineral Resource, Bandung, Indonesia; 5 Faculty of Forestry, Mulawarman University, Indonesia, Samarinda, Indonesia; 6 Faculty of Engineering, Mulawarman University, Samarinda, Indonesia; 7 Fakultas Teknik dan Informatika, Universitas Pendidikan Nasional, Denpasar, Indonesia

**Keywords:** local wisdom, disaster resilience, sustainability, Nusantara capital city, Kalimantan, disaster management, IKN Nusantara, East Kalimantan

## Abstract

Local wisdom in forest conservation underpins disaster resilience in the Mentawir community, enabling effective adaptation to and mitigation of flood risks. Since the village’s establishment, traditional settlement patterns have integrated natural and cultural features that collectively reduce flood exposure. This study aims to revitalise disaster-safety networks that draw on both environmental assets and cultural traditions. Firstly, we examine the role of local wisdom by mapping traditional settlements and their links to cultural practices. Secondly, we assess the sustainability of community engagement and local knowledge in environmental management using multidimensional scaling (MDS), which indicates a relatively weak sustainability status (61.8%). Key drivers of vulnerability include land-use change, economic constraints and infrastructure deficits, all of which require targeted interventions. In response, the Tourism Awareness Group (*Kelompok Sadar Wisata, Pokdarwis*) of ‘Tiram Tambun’ is advancing a community-based economy that safeguards environmental and cultural values through eco-cultural tourism and by promoting cross-regional coordination among management and protection agencies.

**Contribution:**

This study reveals the Mentawir community’s environmental management has relatively weak sustainability (61.8%) based on MDS analysis, prompting the proposal of eco-cultural tourism as a targeted revitalisation strategy.

## Introduction

Situated in the coastal area of Sepaku District along Balikpapan Bay, Mentawir Village is home to the *Paser Balik* people (Badan Registrasi Wilayah Adat 2024), who traditionally sustain themselves through fishing and swidden (slash-and-burn) agriculture. While the community has historically adapted to natural hazards in these lowlands through indigenous wisdom, external pressures intensified in the 1930s with the arrival of migrant groups and a shift in governance under the Kutai Kartanegara Sultanate. As the population grew, residential and agricultural lands expanded to accommodate the region’s increasingly diverse demographic. Today, large-scale upstream mining threatens this traditional equilibrium. This research investigates how Mentawir’s historical resilience and cultural identity can offer valuable lessons for the planning of the neighbouring capital city, Ibu Kota Negara (IKN) Nusantara. Mentawir was settled for its strategic proximity to the sea and freshwater sources; its name – believed to be derived from the word ‘*Penawar*’ [healer] – reflects the river’s historical role in indigenous medicinal practices. The community maintains ecological balance through traditional knowledge, employing nature-based flood defences such as riparian bamboo buffers and estuary mangrove conservation. Regulated timber harvesting, restricted primarily to sailboat construction, exemplifies generations of adaptive practices common among global indigenous communities where local wisdom is vital for mitigating disaster risks (Dhungana et al. 2023). This expertise, refined through long-term ecosystem interaction (Hos et al. 2021), has evolved into sophisticated strategies for managing natural systems and addressing disaster impacts (Šakić Trogrlić et al. 2022). Consequently, global policy frameworks increasingly recognise such local wisdom as a critical resource for climate change adaptation. According to local folklore, the strategic setting of Balikpapan Bay – a nearby bustling commercial entry point – attracted outsiders seeking to exploit Mentawir’s timber. In response, the indigenous Paser Balik sank encroaching vessels, which over time formed artificial reefs known as ‘Tiram Tambun’ (piled oysters). This active defence, combined with the preservation of old forests [*Lati Tuo*], bamboo and mangroves, successfully mitigated flood disasters and defined the community’s coastal identity. However, post-independence modernisation has triggered significant environmental degradation, similar to global trends in China, where traditional landscapes are threatened by rapid urbanisation (see Li & Hu 2019). While other nations have implemented formal revitalisation for historical villages since 2003, the Mentawir community continues to struggle against industrial pressures that jeopardise their traditional settlements. Mentawir’s environmental degradation began with 1960s logging and intensified in the 1990s when PT Inhutani I shifted focus towards industrial rubber and palm oil. This transformation, coupled with the catastrophic 1997 massive forest fires, fundamentally altered the social structure by shifting the community from subsistence farming to industrial labour. Consequently, traditional fields and ancestral mangroves were neglected or even destroyed to support the needs of the charcoal distribution chain, leading to chronic flooding and ecological issues similar to those in traditional Chinese villages (see Sun et al. 2022). As human-induced risks escalate and generate broader repercussions (Mitra & Shaw 2023), the resulting tidal damage to residential areas signals a critical decline in the community’s inherited disaster resilience and traditional adaptive strategies. This research introduces a culturally grounded approach to flood disaster preparedness, aligning with global initiatives that utilise local wisdom for rural revitalisation (Raheem et al. 2023). By examining settlements with deep historical adaptation, the study addresses the diminishing effectiveness of inherited ‘Traditional Ecological Knowledge’ (TEK). While TEK provides a vital empirical foundation for predicting environmental change, a significant barrier remains the entrenched dichotomy between local and scientific knowledge systems (Berkes 1993; Šakić Trogrlić et al. 2022; Stevenson 1996). This paper argues that integrating these two systems is essential for developing comprehensive, resilient disaster management strategies. Moreover, while indigenous practices at the upstream Wain River Protection Forest have demonstrated a significant impact on forest sustainability near IKN Nusantara (Geria et al. 2025), no comparable study has yet addressed the unique coastal environment of Mentawir. To evaluate this integration, the study employs multidimensional scaling (MDS) analysis to assess the sustainability state of current management practices and identify obstacles to resilience. Supplemented by Focus Group Discussions (FGD), the findings highlight a growing community interest in ‘green industry’ initiatives – such as eco-cultural tourism in the *Lati Tuo* [old forests] and mangroves – that balance economic benefits with ecosystem preservation. Ultimately, this research provides scalable recommendations for incorporating local wisdom into disaster management for vulnerable regions.

## Research methods and design

### Research location

This research was conducted in Mentawir Village, Sepaku District (1.0218° N and 116.770° E). It spans 22 222 hectares of lowlands, hills, and mangroves. The study analysed three primary environments: Residential areas, bamboo forests, and protective mangrove buffers. Using purposive sampling protocols (see Sugiyono 2021), 60 respondents – including farmers, labourers, homemakers and civil servants – were selected. Among the farming community, this sample size reflects the population’s homogeneity, which justifies flexibility in participant numbers (Soekartawi 1995). Data were gathered through primary field observations and direct interviews, supplemented by secondary documentation of the region’s physical and social conditions, including the observations previously detailed in the Introduction.

### Data analysis

This study utilises a qualitative methodology to explore the potential of local wisdom within the socio-cultural context of the community. Primary data were collected through focused group interviews, guided discussions and direct observations. The research investigates various elements, including ideology, belief systems, mythology, social structure, regulations, community systems and traditional organisations. It also examines the infrastructure and diverse forms of adaptation to the environment. Secondary data – serving as supplementary information – were gathered from sources within Mentawir Village. Additionally, information was sourced from journals and research reports (printed and electronic media, including the Internet), as detailed in the references. The variables of this study are categorised into three components based on socio-cultural theory (Sanderson 2000): Superstructure, social structure and infrastructure. The superstructure encompasses the ideologies, beliefs and spiritual values that are integral to the community’s culture. The social structure refers to the interactions among individuals within the society, including the established rules, institutions and legal frameworks that govern their behaviour. Lastly, the infrastructure component pertains to the physical facilities and resources utilised by the community. The specific attributes of this research are outlined as follows.

### Superstructure component

The role of the superstructure component in conservation

- The presence of local beliefs regarding the sacredness of disaster-prone environments.
- Traditions of taboos [*pamali*] and the sustainable utilisation of natural resources that contribute to environmental protection.
- Community awareness regarding the preservation of forest ecosystems and protected timber.
- The implementation of rituals associated with the exploitation and use of natural resources.
- The enforcement of local regulations aimed at protecting areas and managing natural resources effectively.
- Community participation in environmental protection initiatives.

### Infrastructure component

The role of infrastructure component in conservation

- The availability of land designated for agricultural purposes.
- Sustainable practices in the utilisation of natural resources, emphasising conservation.
- Traditional management systems for resource allocation and use.
- Effective management of land and hydrological resources.

The sustainability analysis is conducted in several stages, beginning with the identification of attribute functions in management grounded in local wisdom. This framework encompasses six dimensions: ecology, economy, socio-culture, human resources, technology infrastructure and legal institutions. The ordination process employs modified RAPFISH (Rapid Appraisal for Fisheries) software (Kavanagh & Pitcher 2004) to evaluate the regional context (Budiharsono 2005). Each attribute is assessed using an ordinal scale based on sustainability criteria relevant to each dimension, followed by ordination analysis utilising the MDS method. This process includes the development of indices and an evaluation of the sustainability status of existing management functions, analysed both overall and within each dimension (Fauzi 2004).

According to Nurmalina (2008), the MDS technique allows for the mapping of similar points or objects to a single point nearby, while distinct objects or points are represented as farther apart. The score values for each attribute generate a matrix *X* (*n* × *p*), where *n* represents the number of regions and their corresponding reference points, and *p* denotes the number of attributes employed. These values are subsequently standardised for each attribute, ensuring uniform weights across all attributes and eliminating discrepancies between measurement scales.

### Multidimensional scaling

The stages of multidimensional scaling are outlined below:

- Define dimensions and attributes based on the specific issues at hand.
- Weight the data obtained from opinions and real conditions in the research area using pairwise comparison based on the importance scale proposed by Saaty (1993) for comparisons among dimensions and assigning score values to each variable or attribute.
- Establish primary reference points for both good and bad attributes by scoring each variable accordingly.
- Create two additional reference points: A ‘midpoint’ representing a poor score and a point representing a good score. These reference points serve as vertical directional indicators (‘up’ and ‘down’).
- Identify additional reference points known as anchors that can assist in interpreting the ordination results.
- Conduct ordination for all attributes across each dimension. The results are visualised along the horizontal and vertical axes; through a rotation process, the positions of the points can be represented on the horizontal axis, with sustainability index scores ranging from 0% (bad) to 100% (good).
- Conduct the standardisation of score values for each attribute, using Equation 1.

$$X_{iksd}=X_{ik}-X_{k}\cdot S_{ik}$$ [Eqn 1]

where:

*X*_*iksd*_ = Standardised score value for the area (including reference points) for each attribute *k* = 1, 2, …, *p*;

*X*_*ik*_ = Initial score value for the area (including reference points) for *i* = 1, 2, …, *n* for each attribute *k* = 1, 2, …, *p*;

*X*_*k*_ = Mean score value for each attribute *k* = 1, 2, …, *p*.

Based on field observations, calculations, analyses and available secondary data, each attribute is assigned a score or ranking that reflects the sustainability of the corresponding management dimension. These scores represent a ‘bad’ value at one end and a ‘good’ value at the other. The ‘bad’ value indicates conditions that are least favourable for the sustainability of management functions, whereas the ‘good’ value signifies the most favourable conditions. Between these two extremes, there may be one or more intermediate values depending on the number of rankings assigned to each attribute. Subsequently, the score values for each attribute are analysed multidimensionally to identify one or more points that reflect the sustainability status of the management practices under investigation, concerning two reference points: The ‘good’ point and the ‘bad’ point. To facilitate the visualisation of these positions, ordination analysis is utilised. This analysis can also be conducted for a single dimension by incorporating all attributes relevant to that dimension. The results will indicate the degree of sustainability associated with each dimension. Once the analyses for each dimension are completed, a comparative analysis of sustainability across dimensions can be performed and visualised using a kite diagram (Fauzi 2004). The sustainability index scale for local wisdom management functions ranges from 1% to 100%. A function is deemed sustainable if the system being examined achieves an index score greater than 75% (> 75%). It is classified as weakly sustainable if the index score exceeds 50% (> 50%). A function is considered less sustainable if the index score exceeds 25% (> 25%). Finally, it is regarded as unsustainable if the index score falls below 25%. Furthermore, the sustainability index for local wisdom functions will be designated as Institut Kemahiran Baitulmal (IKB-KL), which stands for the Local Wisdom Index. In the next phase, a sensitivity analysis will be performed to identify which attributes contribute most significantly to the IKB-KL at the study site. The impact of each attribute will be evaluated based on changes in the ‘root mean square’ (RMS) ordination, particularly along the x-axis of the sustainability scale. A greater change in RMS resulting from the exclusion of a specific attribute indicates its significant role in shaping the IKB-KL value on the sustainability scale. In other words, the sensitivity of an attribute in determining the sustainability of management functions through local wisdom at the study site is directly correlated with the extent of its influence. Qualitative data for this study were collected via interviews and FGDs, which were audio-recorded with participants’ consent and transcribed. The thematic analysis involved a systematic process of text familiarisation, open coding and iterative theme development. To establish coding reliability, the authors analysed the material independently. This study received ethical approval from the Indonesian National Research and Innovation Agency (Badan Riset dan Inovasi Nasional [BRIN]), and all participants provided informed consent.

### Ethical considerations

Ethical clearance to conduct this study was obtained from the National Research and Innovation Agency (Ref. No. 053/KE.01/SK/01/2024).

## Results

### Natural environmental potential and cultural wisdom of Mentawir in disaster resilience adaptation

Mentawir has a long history of facing natural disasters, especially floods. Over generations, the community has developed a deep understanding of these hazards and put in place culturally rooted strategies to adapt and reduce their impacts. These include early warning systems based on natural indicators, traditional construction methods designed to withstand seismic activity, and water management practices that address both flooding and drought. The community has also established community-led disaster preparedness plans and emergency response protocols. Together, these forms of ecological engineering reflect a holistic approach to managing the environment informed by cultural knowledge. Although Mentawir Village has geological vulnerabilities that may make it less than ideal for settlement, the location is chosen for practical reasons: Convenient access to Balikpapan, rivers that provide a year-round supply of clean water and readily available raw materials for boat-making from the nearby Mentawir forest. Consequently, the Mentawir community demonstrates sustained environmental stewardship, safeguarding ancestral territories and riverine systems along their full course – from headwaters to downstream reaches. Grounded in inherited local knowledge, these practices preserve the integrity of vital resources and reduce exposure to landslides, floods and related hazards (see Table 1). The community’s mitigation measures prioritise preparedness, continuous monitoring and capacity development to anticipate, absorb and recover from disruptive events. Ecological engineering – especially the stewardship of forests in the upper Bengkirai Hills – plays a key role in this effort. These forests are critical to the hydrological system that sustains the Mentawir River. By preserving them, the community helps regulate water flow at the source and protects soil from erosion, thereby preventing rapid sedimentation. Research also shows that many elements of local wisdom in sustainable forest management persist in current practice (Hos et al. 2021). The plant species selected for riverbanks and living fences are chosen for their ability to resist erosion during heavy rain. This approach serves as conservation and land restoration that meaningfully reduces disaster risk. Within shifting cultivation systems, mitigation is supported through land and crop restoration. Although shifting cultivation with slash-and-burn practice remains common, community members are getting used to planting teak, pine and coconut before leaving old fields – both to mark ownership and to promote forest regeneration and soil recovery. The overarching goal is to sustain ecosystem functions and maintain ecological balance. In addition, reforesting logged areas and allowing them to develop into *Lati Tuo* [old scrub]functions as disaster mitigation by helping prevent floods and landslides (see Figure 1). Another ongoing initiative is the creation of a green belt along the riverbanks. This effort preserves riparian forest ecosystems and is complemented by bamboo planting to reduce flood risk – especially important because the village lies in a valley at nearly the same elevation as the river. Bamboo, a tropical species native to Asia, is an effective, locally available option for preventing riverbank erosion. Its dense, fibrous root system binds soil and stabilises slopes and other vulnerable areas. Bamboo culms can also store water during the dry season, which later infiltrates the ground and helps sustain springs. Beyond erosion control, bamboo supports soil microbial communities, increases biomass and contributes to local livelihoods. This approach aligns with Eco-Rejuvenation Technology (ERT) for reclamation and ecology (Singh et al. 2020). As a renewable resource, bamboo supports a variety of crafts and provides social, economic and environmental co-benefits (Thuy et al. 2021). Furthermore, Bamboo cultivation in this region is also guided by Paser customary practices, which include taboos that must be respected – for example, prohibitions on consuming wild deer and yellow bamboo shoots (sprouts). Violations are believed to cause serious illness among offenders.

**FIGURE 1:**
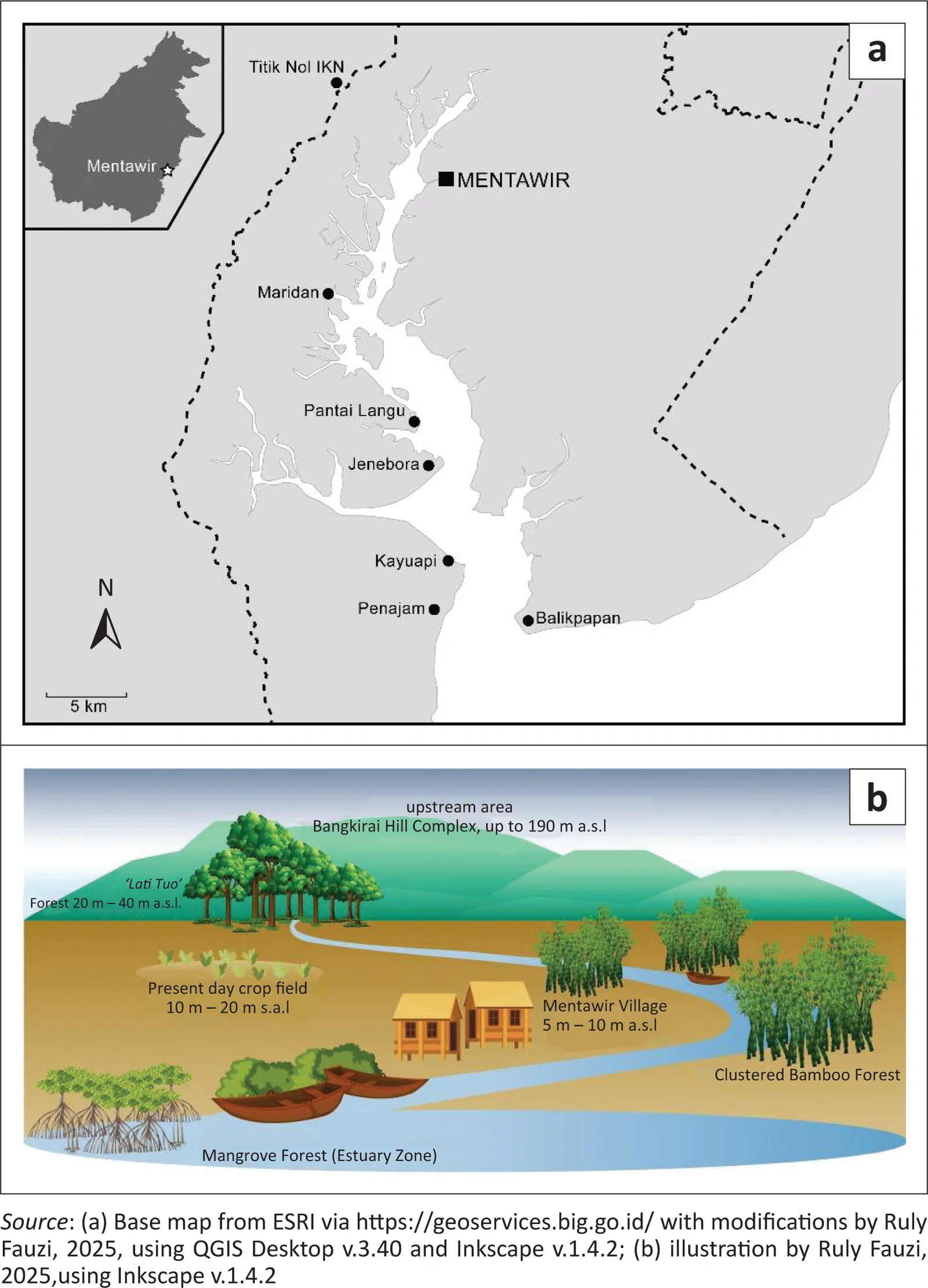
*Lati Tuo* [old forest], bamboo forest and mangroves are actively protected by the Mentawir community to sustain the hydrological cycle and serve as a green belt, protecting against landslides and floods.

**TABLE 1:** Local wisdom and traditional knowledge for sustainable disaster management in Mentawir.

No.	Natural and environmental protection	Associated cultural wisdom	Disaster resilience and mitigation efforts	Role of sustainability and ecological mechanisms
1	*Lati Tuo* [Old Forest]	Soyong	Efforts to safeguard forests against floods and landslides. Prohibition on shifting cultivation. Appeals and invitations to the Indigenous community. Fields previously used for shifting cultivation are designated as protected *Lati Tuo*.	Maintenance of the hydrological cycle and ecosystem integrity
2	Respect for natural rulers and Ancestors	Soyong	Promotion of social and environmental order. Practice rituals as a community.	Fostering harmony within the community and environment
3	Requests for protection from nature and ancestors	Balian	Prayers for protection from harm. Practice rituals as a community.	Valuing ancestral teachings to ensure the safety of nature and environmental harmony
4	Village Head’s command to protect the environment	*Nondoi* and community cooperation [*Gotong Royong*]	Ensuring cleanliness in the village and protection from negative influences. Regular cleaning activities conducted by the community	Cultivating community awareness to establish cleanliness as a norm
5	Reconciliation of conflicts among tribe members	Mayar Salah	Social and customary sanctions addressing community issues and environmental violations, such as illegal logging. Transparent process of *Mayar Salah* custom. Practicing equality and justice among the community members.	Preserving harmony between humans and the environment
6	River preservation	Prohibition against polluting rivers (taboo [*pamali*])	Conservation of rivers and their capacity to manage water flow. Community engagement through collective action. Construction of river diversions. Belief in *Pamali*.	Clean rivers protect aquatic ecosystems and biodiversity.
7	Mangrove conservation	Rituals for the preservation of mangroves. Eco-tourism awareness initiatives	Protection against tidal flooding through mangrove ecosystems. Community-led mangrove management that safeguards local ecosystems. Initiatives aimed at developing the area as a tourism destination, with mangroves also serving as fish breeding grounds.	Mangroves serve as vital habitats for spawning fish and crabs, supporting biodiversity. To enhance local economies through conservation efforts
8	Bamboo forest conservation	Prohibition on exploiting bamboo shoots (taboo [*pamali*])	Protection against river floods. Belief in *Pamali*.	Bamboo’s flexible nature helps retain soil aggregates, serving as a renewable resource.
9	Land stewardship	Gaining community awareness for common disasters. The construction of homes that follow natural contours.	Use of stilt houses for flood protection, employing ironwood for structural pillars. Observing Natural Signs and Tidal Fluctuations. Use of ironwood pillars for enhanced water resistance. Observing Natural Signs and tidal fluctuations. Community elders inform residents to remain vigilant during disasters.	Prevention of landslides through appropriate land use and gaining awareness for flood disasters.
10	Vegetation stewardship	Selective harvesting of wood for boat construction	Boats function not only for transportation but also as crucial tools for disaster mitigation during floods. Guidelines for tree and timber harvesting. Efforts to maintain the sustainability of timber availability by selective logging.	Ensuring sustainable harvesting practices by selecting older trees, thereby maintaining ecosystem balance

Mentawir Village is situated in a bay area that frequently experiences significant flooding because of tidal surges during the rainy season, often compounded by river flooding. To mitigate these impacts, local efforts focus on establishing a mangrove green belt along the village’s seaward boundary. The mangroves thrive here because the village sits at a river mouth that delivers nutrient-rich sediments, supporting vigorous growth. The mangrove forest is also a vital livelihood base, providing spawning grounds for fish and mangrove crabs. Beyond its economic value, the mangrove ecosystem reduces environmental hazards by acting as a natural barrier against coastal erosion and flooding. Empirical evidence shows that mangrove stands – for example, a five year old Rhizophora forest 1 km wide – can reduce coastal wave energy by 72% – 97%, functioning as both a wave buffer and a soil stabiliser. Forest width is a critical determinant of effectiveness, with a minimum threshold of 300 m (Maza, Lara & Losada 2021). A notable local feature is an approximately 100 m^2^ patch of mangroves with yellow leaves, which – according to Paser Balik folklore – originated from the sweat of their ancestors. This belief reflects ecological wisdom, reinforcing the protection and preservation of mangrove genetic diversity for future generations. While mangroves have long been safeguarded by customary norms, some fuelwood harvesting occurred historically. This activity has largely ceased following targeted education and outreach that underscored the ecological importance of mangrove ecosystems. Reflecting Mentawir’s conservation commitments, the village secured financing from the Forest Carbon Partnership Facility (FCPF) under the World Bank’s Carbon Incentive Fund to enhance mangrove management practices. Subsequent government-facilitated trainings enabled local value-addition through processing mangrove fruit into various products: Syrup, coffee blends, traditional sweets [*dodol*] and cooling powder. These products now serve as popular souvenirs in Balikpapan, with Mentawir recognised as one of its local primary sources. The processing is currently coordinated by the Tourism Awareness Group (*Kelompok Sadar Wisata, Pokdarwis*), namely ‘*Tiram Tambun*’, led by Mr. Lamale (age 67 years old). The processing initiative was initiated by PT Inhutani in 2015.

### Ecological engineering through local community belief systems

Local knowledge is empirically grounded, developed and applied over millennia to guide decision-making (Alessa et al. 2016). However, identifying and generalising indigenous practices that support natural resource and ecosystem management is often difficult. Practices may be well documented in one social group but not in another or may be evident in one time period but absent in another. Moreover, researchers without ecological training may overlook or misinterpret practices with ecological significance. As a result, many traditional management practices remain unrecognised for their role in sustaining resources and ecosystems (Folke et al. 2010). Despite the predominance of Islam in the Mentawir community today, local ecological wisdom remains evident. This is because the community continues to respect ancestral beliefs that carry ecological knowledge, rooted in a spiritual relationship between indigenous peoples and their land. Such knowledge is typically passed down orally across generations through cultural traditions such as storytelling, songs and ceremonies (Moncada 2018). The majority of these traditional beliefs are oriented toward soliciting blessings from the natural world and ancestral spirits to maintain social–ecological harmony. Harmony is pursued through sustainable engagement with nature, drawing on resources only as needed. Ritual practice takes the form of ceremonies that employ incantations. While few individuals now retain the capacity to recite these incantations in their original form, most employ the contemporary local language for broader accessibility. Certain customary leaders nevertheless preserve proficiency in the originals. A particularly salient incantation is ‘*Soyong*’, employed by the Paser to invoke divine protection mediated by ancestral spirits. ‘*Soyong Perjalanan*’, recited prior to travel, functions as a normative reminder to exercise care and avoid extractive or careless resource use. The practice also extends to the Paser communities of Penajam–North Paser, where ‘*Soyong*’ is performed with reference to the ancestor Dato Rundad, regarded as the mystical guardian of the region (Mustikawati 2020). In this context, travellers conduct besoyong – reciting incantations to seek Dato Rundad’s permission – when traversing his domain. An illustrative besoyong chant for entering unfamiliar territory follows:

*‘Kampung pana, anak itak koko Kain bayu sumba po kampong. Po kampung ndo Kain ditegur. Takut salak langkah, salah besura Salak alek kanu ulun. Mohon kain diarahkan.’*

[Please remind us if we take a wrong step or if we make a wrong choice. Please, kindly correct and guide us.]

This chant reflects the community’s belief in the presence of guardian spirits, known as ‘*penunggu*’, in specific areas. These guardians, referred to as ‘*Datu*’ or ‘*Nenek*’ [ancestors], are acknowledged through such incantations when someone enters unfamiliar territory. By requesting permission from the local guardian, the individual seeks protection during their time in the new land. ‘*Soyong*’, recited during the ‘*Nampa Ponta*’ ceremony, is performed during the harvest of sticky rice [*padi ponta*]. This incantation serves as an expression of gratitude for the harvest during the community’s harvest festival. Both the ‘*Soyong Perjalanan*’ and the ‘*Nampa Ponta*’ ceremony function as prayers for safety, seeking protection from misfortune and blessings for their efforts. One of the *Soyong* rituals is conducted to seek permission for the utilisation of natural resources while prioritising environmental safety against potential disasters, including flooding. The following is an example of the village elder – a highly influential traditional leader – reciting the *Soyong* incantation, urging the community to safeguard their environment from the source to the estuary:

*‘Aku ini mau ada tujuan, minta ke muara sampai ke hulu, minta dijaga, agar tidak ada permasalahan.’* [I have a purpose; I ask for protection from the estuary to the source, so that no issues may arise.]

Beyond oral traditions, the Paser community adheres to ‘Buyan Bunga Nyora’, a custom legal framework implemented in Mentawir. Sanctions for violations are bifurcated into cultural penalties (for breaches of socio-cultural norms) and natural penalties (for environmental transgressions). Together, these provisions exemplify a culturally grounded form of ecological engineering aimed at preserving forested domains, with emphasis on *Lati Tuo* [old scrub]. Governance is vested in traditional leaders [*Puaq*] and their deputies, whose enforcement roles are essential to maintaining the integrity and sanctity of these areas (Akhmar et al. 2021).

### Adaptive anticipation of ecological engineering through the development of traditional houses

The adaptive capacity of rural settlements is central to socioecological resilience and sustainable development (Sun et al. 2022). In Mentawir, this adaptability is evident in both everyday practices and settlement design. Traditional houses are raised on stilts above flood-prone ground, with roofs engineered to withstand heavy rainfall and strong winds. The village is organised around a communal space known as ‘*Lamin*’, which functions as a gathering place and cultural hub. The surrounding landscape is intentionally curated: trees are planted to provide food and other resources while serving as natural barriers against floods and environmental threats. Stilt houses use ironwood for structural columns to reduce flood damage, and building layouts follow the land’s natural slope to preserve topographic integrity. Socially and culturally, local beliefs about protecting these areas further enhance environmental safety, reinforcing a settlement pattern closely aligned with the region’s natural hazards.

### Sailboats

Mentawir’s sailboats are mostly locally owned and built, serving both livelihoods and flood-time mobility. Two artisans – Mr. Saparudin and Mr. Dulaman, Paser relatives from *Sepaku Lama* [Logdam] – still craft traditional plank-built boats, a legacy that helped make Sepaku a boatbuilding hub. Historically, these boats supported fishing and carried passengers (≈ 3–7 people) and produce to Maridan, Penajam and Balikpapan, where middlemen bought goods. A contraction in boatbuilding is linked to diminished availability and rising prices of quality timber. As reported by Mr. Bahrun (62 years old), a Paser woodcutter, supplies are now largely obtained via building-supply outlets, with much of the wood originating from the upper Mahakam River. The combined effects of Izin Usaha Pemanfaatan Hasil Hutan Kayu (IUPHHK) allocations to PT Inhutani I and the 1997 forest fires have precipitated a significant local shortage of hardwood suitable for boat construction.

This engineering initiative has been in place for hundreds of years, and its benefits are evident even during floods, which do not pose a threat to safety or damage settlements. Tidal flooding has often proven advantageous, facilitating the transport of goods as boats can easily access the shore. However, issues arose with the onset of mining activities in the upstream areas that traverse Mentawir Village. Since the commencement of mining operations, the river has become increasingly turbid, siltation has occurred and the frequency of floods has risen. A significant tidal flood recently impacted Mentawir Village, destroying several settlements (see Figure 2). This disaster was largely attributable to the degradation of mangrove vegetation. In response, the local community and leaders have been motivated to revitalise the mangroves. Community engagement has surged, driven by their desire to develop the area as an integrated tourism destination. This initiative not only mitigates disaster risks by preserving mangrove forests but also fosters community-based disaster mitigation efforts, which are cost-effective and promote community independence, thereby supporting sustainable development (Kurniadi 2019). There is a pressing need to integrate local and scientific knowledge in early warning systems aimed at disaster risk reduction (Hermans et al. 2022).

**FIGURE 2:**
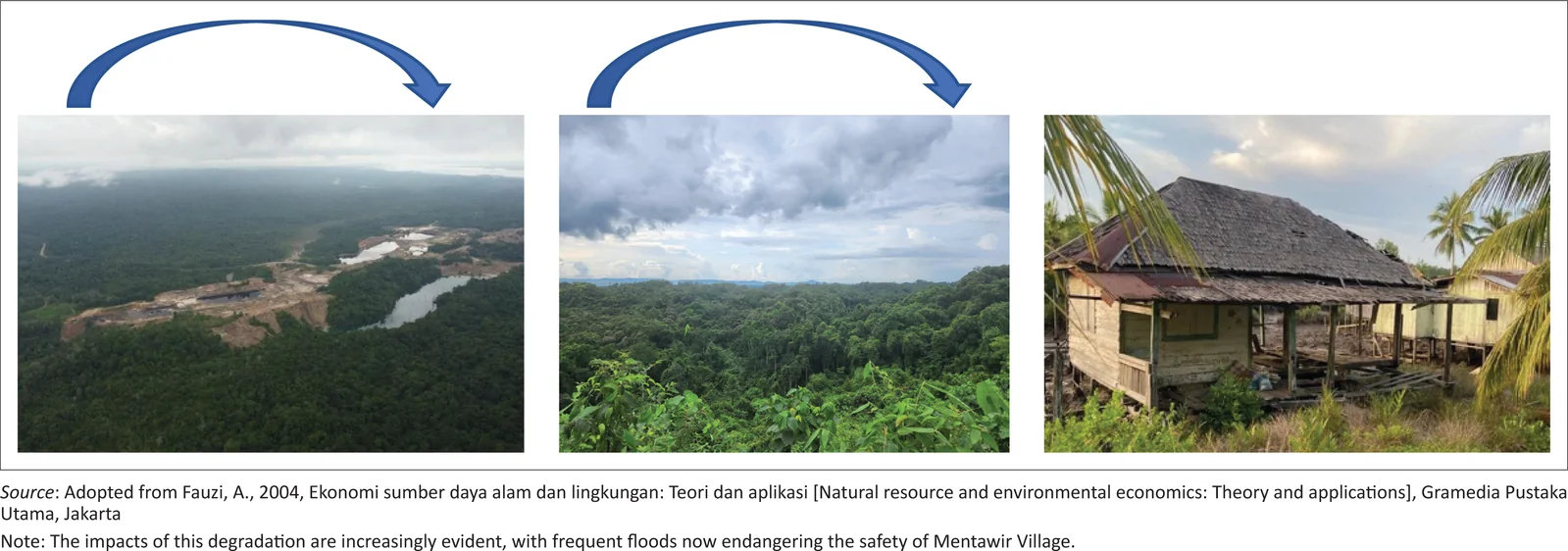
Exploitation in the upstream region poses a threat to the degradation of the old forest (*Lati Tuo*), which has been protected for generations through cultural wisdom to safeguard the hydrological cycle of the Mentawir River.

### Sustainability level of local knowledge and traditional practices for disaster preparedness

In this study, MDS is utilised to map the interrelationships between the Mentawir community’s cultural wisdom and its contribution to disaster resilience. Sustainability is assessed across five key dimensions: *environmental, economic, socio-cultural, technological-infrastructure* and *institutional-legal*. Each dimension comprises 10 specific attributes (variables) evaluated to determine their impact on local knowledge and mitigation strategies (see Figure 3 and Table 2). The MDS analysis indicates that the sustainability of disaster adaptation wisdom in Mentawir Village is currently weak, with an overall score of 61.87%. Below is a breakdown of the sustainability scores across the five key dimensions and the validation of the result:

**FIGURE 3:**
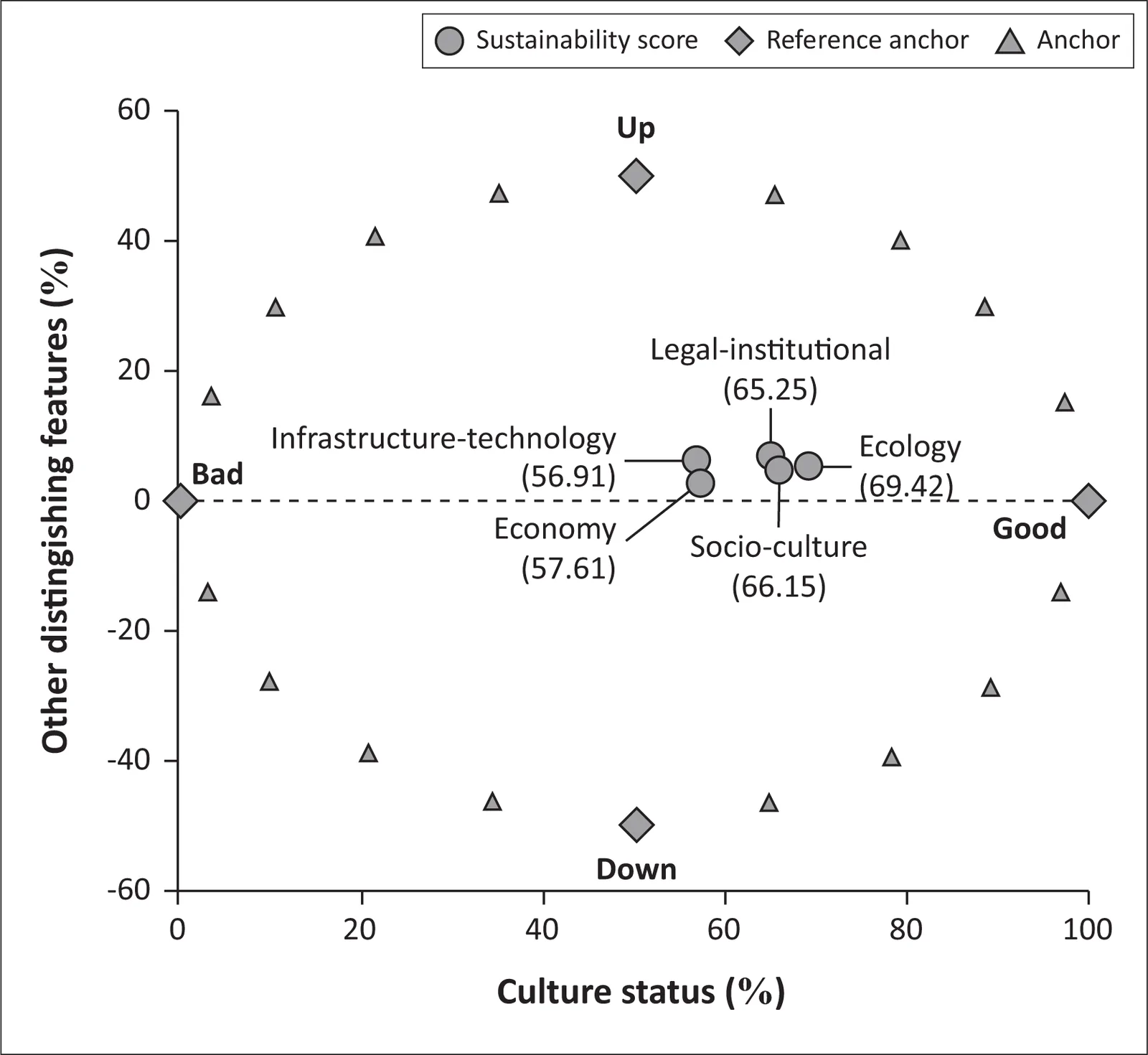
Sustainability scores for each dimension.

**TABLE 2:** Sustainability scores of the five key dimensions observed.

Dimension	Stress value	*R* ^2^	Score (%)	Key sustainability drivers
Environmental	0.25	0.74	69.42	Pressured by logging, mining and land-use conversion.
Socio-cultural	0.23	0.79	66.15	Driven by the continued upholding of traditional customs.
Institutional-legal	0.22	0.80	65.25	Limited by inadequate organisational capacity and customary law recognition.
Economic	0.24	0.69	57.61	Weakened by low added value from sustainable agriculture.
Infrastructure-technology	0.23	0.79	56.91	Hindered by a lack of stakeholder support for green tech.

While the environmental and socio-cultural dimensions strongly support Mentawir’s disaster resilience, they face mounting threats from external factors, such as coal mining and massive plantations. Conversely, critical vulnerabilities appear in the economic and infrastructure-technology sectors (scores < 60%), revealing a ‘sustainability gap’ where traditional values lack the technical support and economic incentives to withstand rapid regional modernisation. Despite sub-optimal MDS stress values of each dimension, the outcomes remain highly interpretable when contextualised by empirical evidence. Moreover, Kruskal’s strict thresholds overlook how larger datasets naturally increase stress because of systemic complexity. However, as Borg (2020) demonstrates, a non-linear relationship exists between data fit and model fit: larger datasets (*n* > 10) can actually improve structural recovery and model accuracy despite higher stress. Ultimately, the contextual interpretability of Mentawir’s shifting landscape outweighs the rigid stress metric itself.

Leverage analysis identified high-influence attributes shaping sustainability scores across dimensions (Figure 4). Two key attributes per dimension were prioritised for improvement. For environmental management and disaster resilience in Mentawir, the main leverage points are:

- Upstream mining that disrupts watershed hydrology.
- Land-use conversion from agriculture to non-agricultural and plantation uses.
- Low farmer incomes relative to the regional minimum wage.
- Price instability driven by limited marketability of mangrove- and bamboo-based products.
- Resource-use conflicts in mangrove and customary forests.
- Low employment absorption from mangrove-based environmental services.
- Degraded irrigation infrastructure upstream and downstream.
- Limited local government resourcing for tidal-flood disaster management.
- Weak regulations and organisational commitment to prevent farmland sales.
- Weak institutional standing and member commitment within community organisations.

**FIGURE 4:**
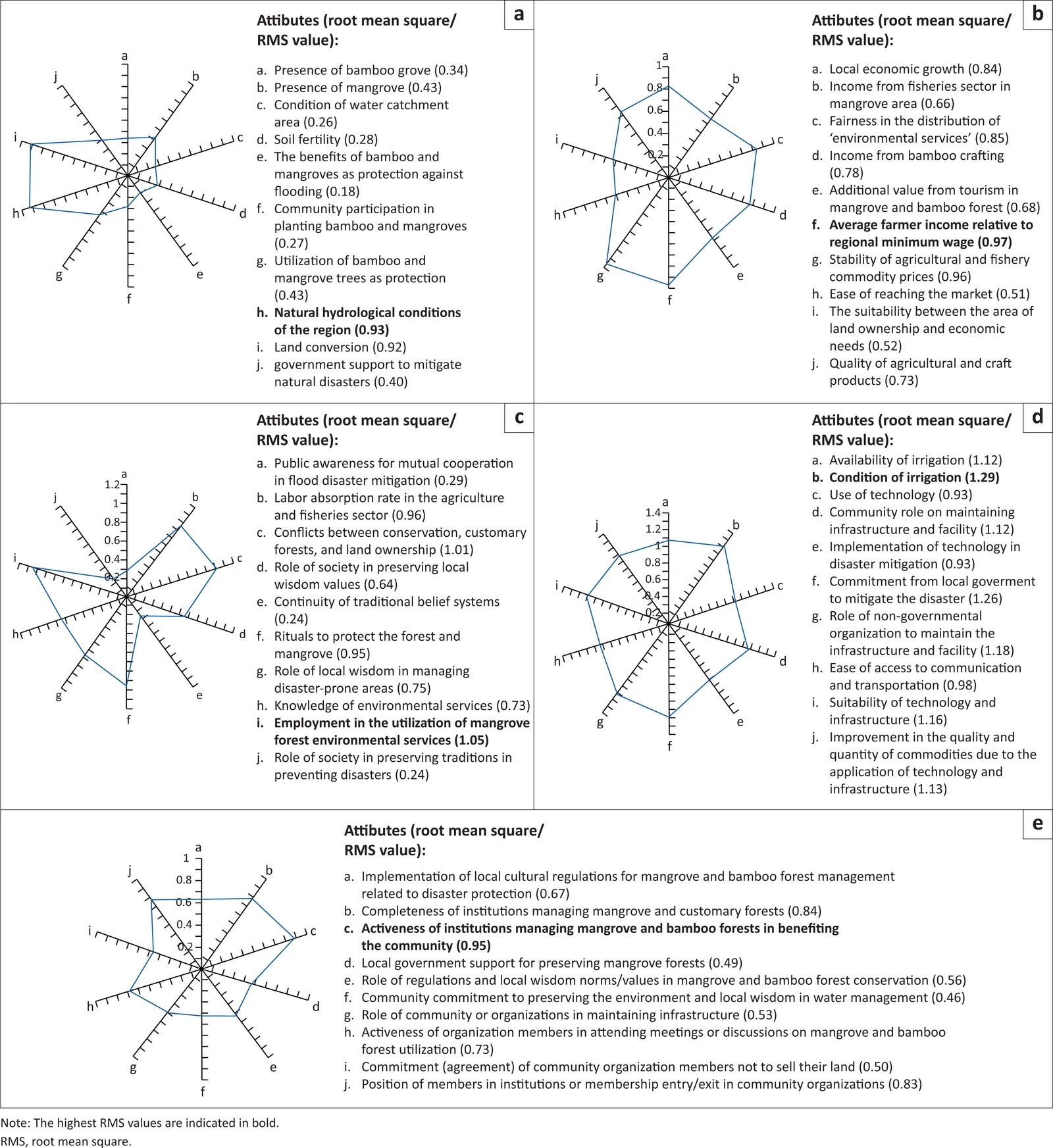
The sustainability scores (leverage) for each attribute from the dimensions of (a) environment; (b) economic; (c) socio-culture; (d) infrastructure-technology and (e) legal-institutional.

The 10 factors outlined above require substantial effort to address. The ecological wisdom inherent in the Mentawir community must be preserved and sustained. Despite significant environmental changes, the local values upheld by the Mentawir people continue to guide their efforts to protect their surroundings – particularly in maintaining resilience against tidal flooding and river overflow. The community’s stewardship of the *Lati Tuo* forest, regarded as a sacred forest, further strengthens its preservation because of the forest’s vital role in preventing disasters.

## Discussion

Observations of Mentawir’s social and cultural dynamics reveal a steadfast commitment to fulfilling ancestors’ mandates to steward a flood-prone landscape. Drawing on traditional ecological knowledge, the community actively engineers its environment by protecting forests, prohibiting shifting cultivation and mobilising collective efforts to conserve river systems. They plant and maintain mangroves to form a green belt that shields the village from floods and cultivate bamboo to mitigate river overflows. Despite shifts in beliefs and religious practice, the community continues to uphold *soyong* – a ritual appeal to nature spirits that exemplifies an ecological-engineering approach to conservation. Rules are enforced through prohibitions or taboos [*pamali*] and social sanctions[*bayar salah*] for those who violate community norms. This constellation of practices reflects a social mechanism that internalises cultural norms through rituals, ceremonies and related traditions. Such rituals help individuals remember rules and interpret signals of ecosystem change. As Chapin (1991) argues, where traditions remain robust, additional measures to preserve knowledge are often unnecessary because people enact culture in practice; similarly, Alcorn and Toledo (1998) show that religious institutions reinforce community cohesion among Indigenous populations.

From a geological standpoint, Mentawir Village is situated in a hazard-prone terrain. Structural geology exerts a primary control on landscape evolution and is expressed at the Earth’s surface; tectonically influenced landscapes are characteristically shaped by folding and faulting (Noor 2010). In the study area, morphological evidence indicates rolling topography and a northeast–southwest lithological structure (Figure 5), defined by thrust faulting intersected by normal faulting and graben development. Regionally, folding is prominent, with well-developed anticlines and synclines. The interaction of slope gradients with clay-rich bedrock increases shear susceptibility and thus the likelihood of landslides. Implementing sustainable forest-management practices can reduce soil erosion, improve slope stability and contribute to the conservation of the landscape. In addition to the hazards posed by potential land movement, eroded and deposited clay formations further complicate local conditions. Because clay has low permeability, it limits the infiltration of surface water into the soil. Clay-rich strata often have high porosity but transmit water poorly, so they drain slowly and promote surface runoff. During the rainy season, when surface flow in the Mentawir area increases, the accumulation of clayey sediment further depresses infiltration capacity, elevating flood risk. Preserving forests is therefore crucial: canopy interception, ground cover and root systems enhance infiltration, reduce overland flow and stabilise slopes, helping to mitigate these natural constraints across the Mentawir region. Empirical evidence shows that forest and freshwater ecosystems help mitigate urban heat risk through transpiration and solar-radiation interception. They also support food security by enhancing evapotranspiration, shortening runoff duration and promoting infiltration. Along coastlines, ecosystems reduce erosion by dissipating wave energy and conserving coastal nutrients that sustain ecological succession. This review highlights that hazard-mitigation effectiveness varies by forest type and spatial extent (Paudel, Dhakal & Sharma 2024), with natural ecosystems functioning as critical buffers against disaster impacts. These processes are increasingly recognised as integral to strengthening community resilience. Moreover, communities’ efforts to adapt by modifying and managing their surroundings reflect an understanding of the inherent hazard exposure of their settlement areas, which are empirically less suitable for habitation owing to these risks. Mentawir is no longer the remote village it once was. The opening of the highway in 1980 began to draw outsiders into the area, gradually transforming the landscape and the lives of those who lived there. As mining industries expanded upstream, new economic opportunities emerged, encouraging many residents to leave behind traditional farming. Yet, this shift also slowly weakened the community’s connection to long-held environmental values. Environmental assessments show that two forces now shape Mentawir’s ecological sustainability: rapid changes in land use and the deteriorating condition of the river basin’s hydrological systems. Farmland that once supported local livelihoods has been converted into mining sites, especially upstream. This transformation has destabilised slopes, triggering landslides and sending heavy sediment into the river. Over time, the river has grown increasingly turbid and shallow, making seasonal flooding during the rainy months far more common. Water scarcity has become one of the community’s most urgent concerns. Abandoned coal mines, left unreclaimed, fill with water that eventually flows into the Mentawir River – the village’s primary source of clean water. Instead of nourishing the community, this runoff carries sediment and pollutants that further degrade the river. What was once a dependable water source now reflects the growing environmental pressures brought by upstream industrial activities. In Mentawir’s economy, two pressures weigh heavily on smallholders: low average incomes and unstable crop prices. Tensions over mangrove use add to the strain. Although sustainable mangrove services could anchor new livelihoods, they have yet to generate meaningful jobs for the community. A shift towards an environmentally sustainable, service-based economy could help preserve indigenous ecological knowledge, but that wisdom risks fading as more residents leave farming for mining. Governance gaps seem to have deepened the problem. Local authorities show limited commitment to managing the river system from upstream to downstream, while community engagement in land-use decisions remains weak. Participation in indigenous governance is also limited, in part because cultural knowledge passed down by elders is no longer widely understood. Even as the community demonstrates strong resilience strategies, unchecked environmental exploitation threatens both their traditional knowledge and their capacity to cope with future disasters. The stakes are rising with large-scale commercial development tied to the Capital City of Nusantara, which is likely to disrupt ecosystems, alter the local climate and accelerate the erosion of traditional practices. Scarce resources and inadequate infrastructure further constrain community resilience amid mounting environmental and social pressures. Though later than ideal, Mentawir has begun to pursue growth aligned with conservation. What is needed now is firm, coordinated action to address the key leverage points undermining traditional management – practices rooted in indigenous wisdom that have proven effective in mitigating floods and must be reinforced for long-term resilience.

**FIGURE 5:**
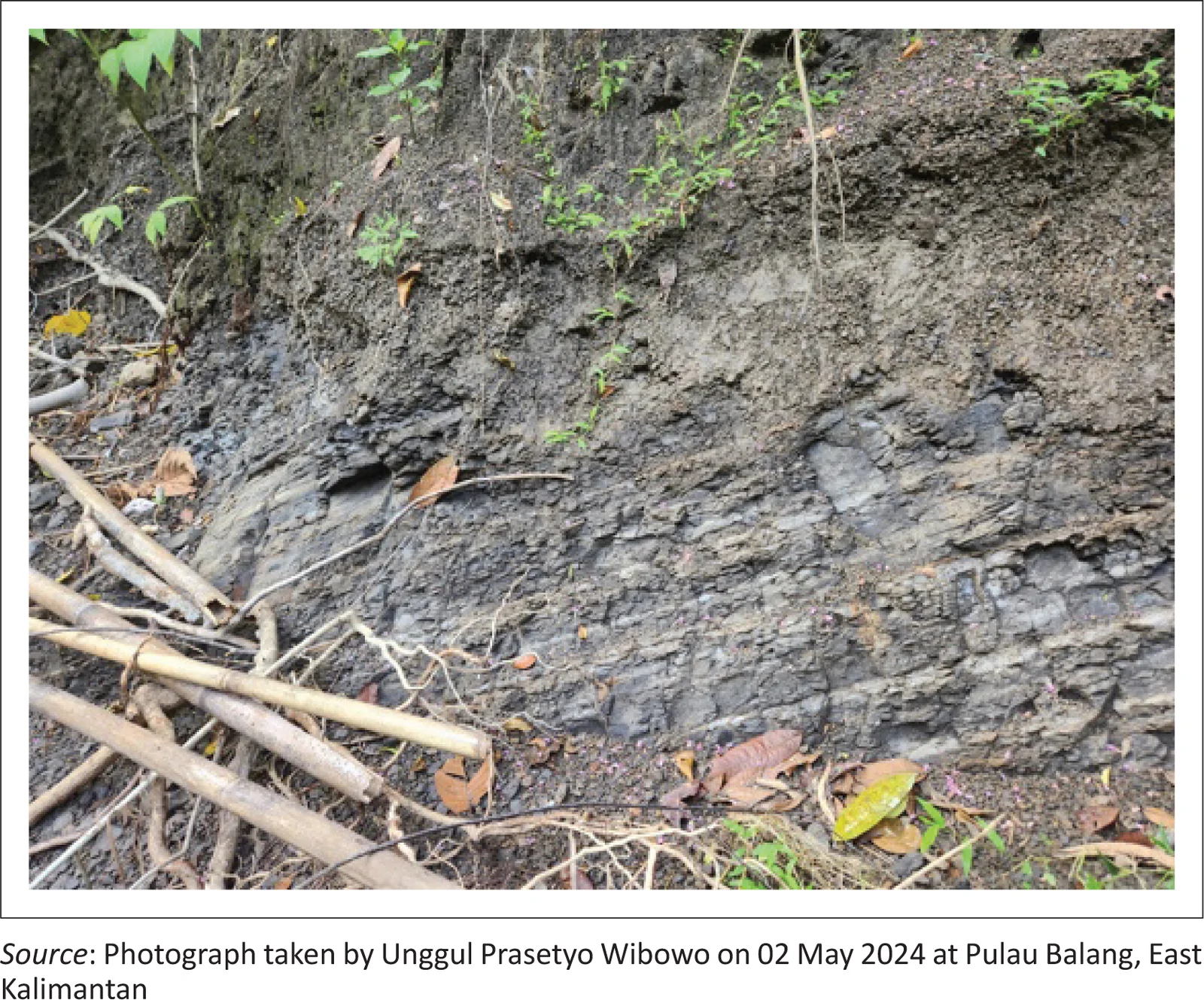
Sedimentary clay rock formations of the Pulau Balang formation, influenced by folding structures, resulting in tilted layers.

## Conclusion

This study demonstrates that the traditional settlement landscape of Mentawir Village is not merely a historical relic but a functional model of indigenous resilience. By synthesising geographical context with TEK, our research highlights a community whose survival is intrinsically linked to its ability to adapt to the volatile coastal environment of East Kalimantan.

The MDS analysis reveals a significant disparity in the village’s disaster resilience. While the environmental (69.42), socio-cultural (66.15) and institutional-legal (65.25) dimensions show robust sustainability, critical vulnerabilities persist in the economic (57.61) and infrastructure-technology (56.91) sectors. This quantitative evidence suggests that while the community’s cultural and ecological foundations remain intact, their resilience is being undermined by a lack of modern technical support and economic diversification. To bridge this gap, a strategic intervention focusing on 10 key leverage variables is essential to transform these latent cultural strengths into a comprehensive disaster-management framework. The Mentawir community’s reverence for nature serves as a vital psychological and practical buffer against environmental hazards. Their proven methods of flood mitigation and resource management represent an invaluable empirical resource. In an era defined by climate instability, preserving this cultural wisdom is a global necessity. We argue that the dichotomy between traditional and scientific knowledge must be dissolved; a resilient future depends on integrating TEK into formal research and capacity-building initiatives. As Indonesia develops its new capital, IKN Nusantara, within this very landscape, a historic opportunity exists to avoid the pitfalls of conventional urbanisation. Mentawir provides a blueprint for ‘blended resilience’ – where the planning and construction of sustainable settlements leverage deep-time local ecological understanding. By incorporating the Paser Balik’s adaptation strategies into modern urban design, the new capital can move beyond Western-centric models to become a global exemplar of a culturally grounded, disaster-resilient metropolis.
